# Evaluation of Bias Correction Method for Satellite-Based Rainfall Data

**DOI:** 10.3390/s16060884

**Published:** 2016-06-15

**Authors:** Haris Akram Bhatti, Tom Rientjes, Alemseged Tamiru Haile, Emad Habib, Wouter Verhoef

**Affiliations:** 1 Department of Water Resources, Faculty of Geo-Information Science and Earth Observation (ITC), University of Twente, Hengelosestraat 99, Enschede 7514 AE, The Netherlands; t.h.m.rientjes@utwente.nl (T.R.); w.verhoef@utwente.nl (W.V.); 2 Department of Civil Engineering, NED University of Engineering and Technology, Karachi 75270, Pakistan; 3 International Water Management Institute, P.O. Box 5689, Addis Ababa, Ethiopia; a.t.haile@cgiar.org; 4 Department of Civil Engineering, University of Louisiana at Lafayette, Lafayette, LA 70504, USA; habib@louisiana.edu

**Keywords:** CMORPH, bias factor, Gilgel Abbey, satellite rainfall correction, optimum window size

## Abstract

With the advances in remote sensing technology, satellite-based rainfall estimates are gaining attraction in the field of hydrology, particularly in rainfall-runoff modeling. Since estimates are affected by errors correction is required. In this study, we tested the high resolution National Oceanic and Atmospheric Administration’s (NOAA) Climate Prediction Centre (CPC) morphing technique (CMORPH) satellite rainfall product (CMORPH) in the Gilgel Abbey catchment, Ethiopia. CMORPH data at 8 km-30 min resolution is aggregated to daily to match *in-situ* observations for the period 2003–2010. Study objectives are to assess bias of the satellite estimates, to identify optimum window size for application of bias correction and to test effectiveness of bias correction. Bias correction factors are calculated for moving window (MW) sizes and for sequential windows (SW’s) of 3, 5, 7, 9, …, 31 days with the aim to assess error distribution between the *in-situ* observations and CMORPH estimates. We tested forward, central and backward window (FW, CW and BW) schemes to assess the effect of time integration on accumulated rainfall. Accuracy of cumulative rainfall depth is assessed by Root Mean Squared Error (RMSE). To systematically correct all CMORPH estimates, station based bias factors are spatially interpolated to yield a bias factor map. Reliability of interpolation is assessed by cross validation. The uncorrected CMORPH rainfall images are multiplied by the interpolated bias map to result in bias corrected CMORPH estimates. Findings are evaluated by RMSE, correlation coefficient (r) and standard deviation (SD). Results showed existence of bias in the CMORPH rainfall. It is found that the 7 days SW approach performs best for bias correction of CMORPH rainfall. The outcome of this study showed the efficiency of our bias correction approach.

## 1. Introduction

Hydrological studies related to rainfall-runoff modeling and flood forecasting require information on precipitation as a major input. Traditionally, information on precipitation is available and collected from ground based meteorological gauging stations. For area coverage, estimates are spatially interpolated to arrive at representation of spatially distributed rainfall fields. Alternative to *in-situ* estimates are space born estimates. Each source has its own advantages and limitations. For instance, space born estimates provide spatial coverage by means of images with pixel sizes of e.g., 1 km^2^ or much larger (>25 km^2^). As such, spatial resolution is low in comparison to *in-situ* observations which are considered point measurements [1] but it is the only available source of dense coverage of precipitation in mountainous region. The spatial coverage of space borne estimates (such as radar) often contains missing data from different causes such as blockage that is specifically applicable in heavy storms [2].

Recent research by Habib *et al.*, Vila *et al.*, and Ebert *et al.* [3,4,5] indicate that satellite-based rainfall estimates are not always reliable and consensus has been reached that estimates require correction. For assessment of reliability and accuracy of satellite-based estimates, observations are compared to *in-situ* measurements by rain gauges that are considered to be the “true” estimate [4,6]. However, rain gauge networks often have low density with stations that are not evenly distributed in regions such as in mountainous and remote terrain. Moreover, rainfall time series are often incomplete or may be affected by erroneous estimates caused by poor maintenance. As such time series are not reliable and may hamper hydrological assessments and modeling studies, this has boosted research on the assessment of accuracy of Satellite Rainfall Estimates (SREs). Considering data scarcity in poorly gauged regions, it is important to assess whether SRE’s are suitable to act as a substitute or supplement data source.

One of the available satellite rainfall data sources is the NOAA’s CMORPH product that uses multiple passive microwave (PMW) satellites and infrared (IR) images for estimation of precipitation (see Section 3.1). Satellite products are mostly prone to errors [1,2,4] as they are estimated from secondary sources (for instance, cloud top brightness temperature). Early in the development of SREs, Rosenfeld and Mintz [7] found that PMW precipitation products are subject to bias due to incorrect measured brightness temperature in semi-arid regions. Smith *et al.* [1] stated that CMORPH biases might be due to diurnal sampling bias, tuning of the instrument or the rainfall algorithm, or unusual surface or atmospheric properties that the instrument does not correctly interpret. Joyce *et al.* [8] applied a Kalman filter approach to reduce bias in the CMORPH estimates and integrate estimates with *in-situ* observations. Studies by Ebert *et al.*, Xie and Arkin [5,9] advocated calibrating the SREs using direct measurements (*i.e.*, *in-situ* stations). We use gauge data as a benchmark for bias correction in the CMORPH estimates. In merging, satellite data with *in-situ* observations, any bias in the satellite data is quantified and corrected so that the resulted estimate is consistent for further use.

Several studies have been conducted to evaluate the accuracy of CMORPH rainfall data. Bitew and Gebremichael [10] assessed the adequacy of a number of high resolution (0.25° × 0.25°, 3 h) SREs as an input to SWAT hydrologic model for daily stream flow simulations. Products tested were TRMM TMPA 3B42RT, TRMM TMPA 3B42, CMORPH and PERSIANN. It is observed that the bias-corrected CMORPH estimate performs better than the PERSIANN and TRMM TMPA 3B42 products. Bitew *et al.* [11] compared the CMORPH, TRMM TMPA 3B42RT, TRMM TMPA 3B42 and PERSIANN products with the *in-situ* rainfall records in a small catchment of the Nile watershed. It is observed that the products underestimated the actual rainfall fields with the lowest underestimation values being observed for the CMORPH and TRMM TMPA 3B42 products. Haile *et al.* [12] used eight automatic rain gauge stations to evaluate the accuracy of CMORPH estimates. It was found that the accuracy of CMORPH estimates varied across the basin area and that estimates did not capture well the rainfall temporal variability. The result showed that CMORPH has weak identifying ability because of which the total bias becomes more than 50% of the seasonal rainfall depth. CMORPH reported that heavy rains are more frequent in lowland areas as compared to *in-situ* stations in mountainous regions. Habib *et al.* [3] in their study showed that bias in CMORPH is dependent on topography and latitude across the catchment of the Nile basin. Their study also depicted that bias factor changes in accordance with seasonal variation, latitude and topography. Dinku *et al.* [13] compared 10 day CMORPH estimates with *in-situ* records and found a correlation coefficient of 0.74 for an Ethiopian catchment. Hirpa *et al.* [14] reported a fair match between CMORPH and TRMM TMPA 3B42 rainfall patterns in an Ethiopian basin.

Leander and Buishand, Shabalova *et al.*, and Terink *et al.* [15,16,17] corrected SREs by dividing time series into different subsets of full data. In these studies, sampling windows of different lengths are applied to subdivide the entire record. Sampling window analysis is usually applied to cater variation in the time series data. In general, sampling window analysis is of two types (i) Moving Window (MW) and (ii) Sequential Window (SW). In the MW approach, the subset is updated by “shifting forward”; that is, eliminating the first number of the series and containing the succeeding number following the original subset. This creates a new subset of numbers that is repeated for the full record. In SW approach, the subset is modified by moving the entire window of the subset, without repetition of numbers in the earlier window.

A number of studies report bias correction of satellite data. Satellite rainfall data has been corrected by gamma transformation [18], but the authors found that the corrected estimates do not capture temporal variability as shown by *in-situ* data. Leander and Buishand [15] corrected the standard deviation of SREs using a regression equation. They applied the SW approach and selected 65 days of sampling windows following Shabalova *et al.* [16] who reported that bias in the satellite data reduces using a 70 day sampling window. Terink *et al.* [17] stated that “length of the sampling window should not be too small, because then one would be correcting for differences which are caused by natural variability instead of correcting for systematic model errors”. In the same study the SW approach applied with block sizes of different lengths on the satellite rainfall of 17 years. It is found that RMSE between observed and corrected data is reduced for a 65 day block length.

Our study is based on the following objectives: (a) to evaluate bias between the *in-situ* (gauge) rainfall and satellite (CMORPH) data; (b) to identify the optimum window size for application of bias correction and (c) to apply bias correction to CMPORPH data. This paper comprises of following sections: the study area is described in Section 2. In Section 3, the datasets are discussed. The approach followed is presented in Section 4 that is further sub-divided in to five subsections: the length of the sampling window, bias correction method, inverse distance weighted interpolation, cross validation and overall assessment of bias-corrected CMORPH estimates. Afterwards, in Section 5 and Section 6, results and conclusions are drawn, respectively.

## 2. Study Area

**Figure 1:**
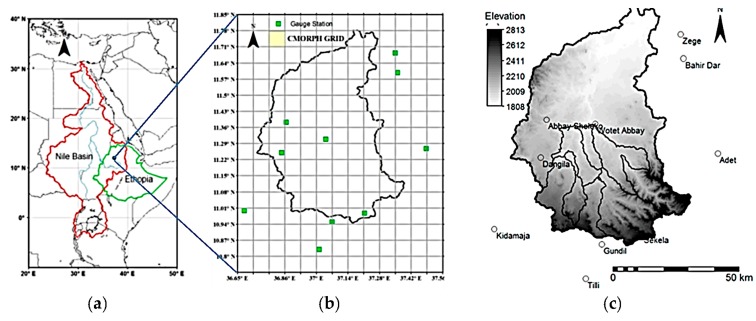
(**a**) Study area depicts location of the Gilgel Abbey catchment across the Nile basin (**b**) Position of ten (10) *in-situ* stations overlain on the CMORPH GRID are marked (**c**) DEM of the Gilgel Abbey catchment.

The focus of this study is the Gilgel Abbey watershed that is one of the major inflow contributors to Lake Tana, Ethiopia [19]. Our study based on the Upper Gilgel Abbey subwatershed in which rain gauge stations have been installed by Ethiopian Meteorological Agency (EMA) and for which streamflow data is available through the Ministry of Water and Energy of Ethiopia. The subwatershed is located between latitudes of 10°56′N–11°22′N and longitudes of 36°49′E–37°24′E (Figure 1). Its catchment area is 1655 km^2^ with elevations that range from 1808 to 2813 m above sea level. It is predominantly covered by agricultural land having clay to clay-loam as a dominant soil type. The seasonal rainfall distribution of Gilgel Abbey is influenced primarily by the rainy season of Intertropical Convergence Zone which matches with the summer of the north hemisphere (June–August).

At short term scales, the rainfall pattern in this catchment is affected by topographical variation and the presence of Lake Tana [20,21]. Heavier rainfall at a short time scale is noticed more frequent in the lowlands than in the highlands of Gilgil Abey [22]. As per the Köppen Classification System [23], the catchment has mild weather with dry winters and warm summers.

## 3. Datasets

### 3.1. CMORPH Satellite Product

In this research study, we selected SREs by CMORPH which is available at 8 km × 8 km resolution and 30 min observation intervals. CMORPH is a grid-based rainfall estimate that mainly relies on multiple PMW sensors from low orbiting satellites and uses IR data from the Geostationary Operational Environmental satellite (GOES) [24]. It combines instantaneous rain rates estimated from different PMW sensors that include Special Sensor Microwave Imager (SSM/I) [25], Advance Microwave Scanning Radiometer-Earth Observing System (AMSR-E), Advanced Microwave Sounding Unit (AMSU-B) [26] and Tropical Rainfall Measuring Mission Microwave Imager (TMI) [27]. NOAA states that CMORPH is a morphing technique that incorporates estimates from existing microwave rainfall algorithms. CMORPH is adaptable enough that rainfall estimates from any microwave satellite source can be integrated through this technique.

Estimates obtained from PMW sensors are in a sequence of hours, similar to the overpass frequency of the low orbiting satellites. Findings from PMW sensors have spatial and temporal loopholes that restrict their suitability for hydrological modeling and water resources assessment. To fill these gaps, the algorithm utilizes cloud motion vectors extracted from spatial lag correlation of successive GOES IR images. Radar rainfall motion is used to further adjust the cloud motion vectors. These altered vectors are used to propagate the PMW-based rainfall estimates for the time periods between two successive PMW overpasses. The shape and intensity of the rainfall pattern is then morphed through linear interpolation using weights that are obtained from forward advection and backward advection of rainfall features [24].

The CMORPH data is available since December 2002 globally (60°N–60°S) at spatial and temporal resolution of 8 km × 8 km and 30 min respectively that makes it suitable for hydrological applications and water resources assessment. Owing to its high spatio-temporal resolution, free availability and being one of the most accurate satellite rainfall estimates [5], CMORPH is selected for this study. We use the CMORPH version 0.x for bias correction. It is a pure satellite precipitation product, produced using only satellite observations and became operational since December 2002 [24]. In this study, we aggregated 30 min CMORPH data to daily time scale to match with *in-situ* observations. Depending on the accessibility of rain gauge data, we selected the CMORPH data covering the period January 2003 to December 2010.

It is to be noted that CMORPH has also released an additional bias-corrected precipitation product (CMORPH 1.0), which uses gauge observations for bias correction. We compared CMORPH version 1.0 with our bias corrected CMORPH product (see Taylor’s diagram in Section 5.5).

### 3.2. In-Situ Rain Gauge Data

In the Gilgel Abbey area, an *in-situ* monitoring network of ten stations is installed by EMA that records daily rainfall data in daily time steps. The network is spread over an area of 110 km × 100 km and used for evaluation of SREs [12,20,22] and hydrological applications [28,29,30,31,32,33]. Figure 1 represents a Shuttle Radar Topography Mission (SRTM) digital elevation model (DEM) of the area with stations position shown by green squares overlaid on CMORPH square grids. Although gauges are unevenly distributed over the watershed, Haile *et al.* [20] showed that gauge locations are appropriate to represent the variability of rainfall over the watershed. As such in this study time series from the network of stations are used as a reference dataset for evaluation and correction of CMORPH rainfall estimates.

**Figure 2:**
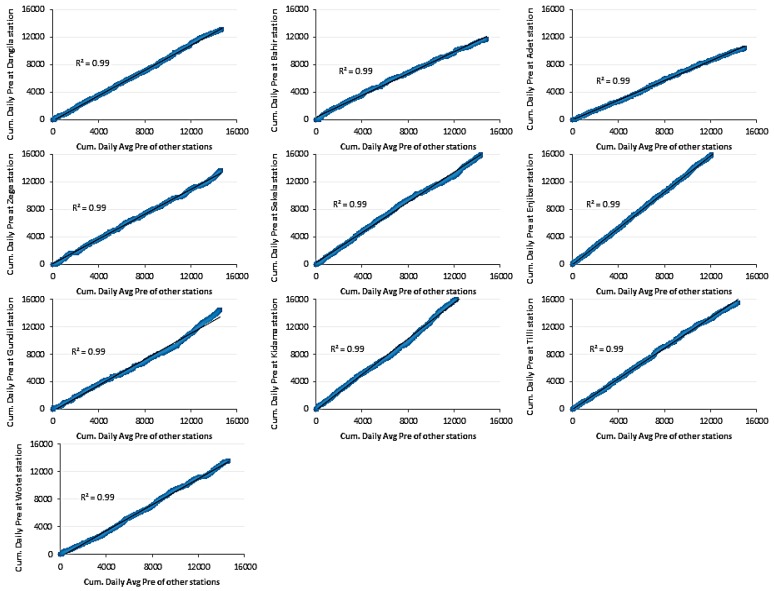
Cumulative daily rainfall time series for each *in-situ* station against cumulative daily average rainfall of remaining *in-situ* stations.

Since the installed *in-situ* stations are limited in number with interstation distances larger than 10 km [12], we applied double mass curve analysis for screening of the *in-situ* data. This analysis ensures that any trend detected in the *in-situ* time series are due to meteorological causes and not to changes in station location or in observational methods. Following McCollum *et al.* [34], the *in-situ* data set is screened to assure its quality before being applied for bias removal in the CMORPH estimates. Consistency of time series is checked by double mass curve analysis by plotting cumulative daily rainfall for each station against cumulative daily average rainfall of an ensemble of remaining *in-situ* stations (see Figure 2). For all plots, the coefficient of determinations (*R*^2^) was found to be 0.99 or higher, which suggests good agreement between each *in-situ* station and the average of the ensemble of remaining *in-situ* stations. High value of *R*^2^ assured consistency of time series rainfall data and suggests that data may be further use for bias correction without applying any filtering. For this study, we considered time series from January 2003–December 2010 according to availability of *in-situ* rainfall data and CMORPH data. Considering the *in-situ* data during the analysis period, the average annual rainfall is found to be ~1620 mm. For detail understanding of the Gilgel Abbey catchment and *in-situ* stations, reference is made to [12,20,22].

## 4. Methodology

### 4.1. Length of the Sample Window

Satellite-based rainfall estimates are commonly affected by random and systematic (bias) errors [35,36]. In this study, we focus on correcting systematic errors. Therefore, samples suitable for correction need to be representative in a way that it captures the natural variability occurring in the rainfall records. Habib *et al.* [37] used time periods (*i.e.*, window) of 7 days for which rainfall was accumulated. In the approach it was assumed that minimum rainfall depth should be 5 mm that resulted from a minimum of 5 rainy days. Terink *et al.* [17] evaluated a bias correction method for the correction of SREs, and tested 25, 35, 45, 65, 85, and 105 sampling windows. SRE is rectified through statistical indices considering 65 days sampling window.

In this study, MWs and SWs of different lengths are applied to calculate the statistics between rainfall estimates by rain gauges and CMORPH. In the MW approach, a time window of specified length moves forward in the time domain on a daily base. In the SW approach, the window moves forward in the time domain by the selected window size. It is to be noted that in this approach a single bias factor is estimated for all days of the selected window. Therefore, this approach is not applicable for FW, BW and CW schemes and thus not tested.

Window sizes of different lengths (3, 5, 7, ..., 31 days) are tested for both approaches. Principle to the selection is that a long window size reduces/smoothens the time series variability at smaller time scales such as ‘day by day’ variability whereas a short window size may result in too low accumulation of rainfall thus, presumably, introducing very low (or very high) or even zero error in case of dry days. Also for the bias calculation, the sampling window should allow adequate rainfall accumulation to incorporate the temporal variation. As such criteria for window selection are: (i) a window covering at least 3 days that will allow sufficient accumulation of rainfall; (ii) a RMSE that does not increase pronouncedly with increase of window size and levels off at the optimum sampling window.

### 4.2. Bias Correction Method

In this study a multiplicative bias factor, also termed multiplicative shift technique [38] is used to correct the daily CMORPH estimates. The ratio between gauge and satellite estimates is calculated that is multiplied by the satellite estimates to arrive at the biased corrected rainfall estimate. We tested MW and SW approaches for removal of bias in the CMORPH estimate. MW approach is tested with three different schemes (FW, BW and CW) to evaluate effects of time integration on accumulated rainfall.

Windows with sizes of 3, 5, 7, 9, …, 31 days are tested for MW and SW approaches and RMSE is estimated to assess error distribution between the *in-situ* observations and CMORPH estimates. The principle of the FW, BW and CW schemes is to reduce discrepancy between satellite and *in-situ* rainfall estimates considering temporal data at the relevant gauge location. For instance, FW scheme of size 7 days represents bias factor for the current day considering CMORPH and *in-situ* estimates of the following six days. Similarly, CW of 5 days shows bias factor of the current day considering rainfall estimates of previous two days and following two days. For analysis, rainfall detection by the satellite is assessed with the corresponding *in-situ* observations overlain on the pixel.

For a certain day d and gauge i the multiplicative bias factor at a specific CMORPH pixel with an overlain gauge can be expressed as follows:

(1) $$BF_{i}^{d}=\frac{\sum_{t=d-m}^{t=d+m}P_{g}\left(i,t\right)}{\sum_{t=d-m}^{t=d+m}P_{s}\left(i,t\right)}$$

where $P_{g}$ and $P_{s}$ represents daily gauge and satellite rainfall amounts respectively at a given gauge/pixel location i and time instant t, m represents no. of days before/after considered in the sampling window such that:

(2) $$m=\frac{l-1}{2}$$

where l is the length of the sampling window. It is to be noted that Equation (1) is applicable to CW schemes and SW approach. It is also to be noted that CW scheme is only applicable to window size of 3, 5, 7, …, 31 days. For FW and BW schemes, Equations (3) and (4) apply:

(3) $$BF_{i}^{d}=\frac{\sum_{t=d}^{t=d+l}P_{g}\left(i,t\right)}{\sum_{t=d}^{t=d+l}P_{s}\left(i,t\right)}$$

(4) $$BF_{i}^{d}=\frac{\sum_{t=d-l}^{t=d}P_{g}\left(i,t\right)}{\sum_{t=d-l}^{t=d}P_{s}\left(i,t\right)}$$

Equations (1), (3) and (4) give a daily BF that differs at spatio-temporal scales. To elaborate the sampling window analysis, we refer to Table 1 in which BF and corrected CMORPH is estimated using MW and SW (FW, BW and CW schemes) approach. The analysis is done for Bahir Dar station and 3 days sampling window.

For further clarification, BF calculation using FW and BW scheme (for day 175 and 176 respectively) is shown below:

$$BF_{Bahir\;Dar}^{175}=\frac{27.8+54.0+16.5}{8.7+6.5+14.8}=\frac{98.3}{30}=3.28$$

$$BF_{Bahir\;Dar}^{176}=\frac{54.0+27.8+1.5}{6.5+8.7+0.6}=\frac{83.3}{15.8}=5.27$$

The above BF’s are estimated using Equations (3) and (4), respectively. For the CW scheme and SW analysis, BF calculation using Equation (1) (for day 176) is shown below:

$$BF_{Bahir\;Dar}^{176}=\frac{27.8+54.0+16.5}{8.7+6.5+14.8}=\frac{98.3}{30}=3.28$$

It is to be noted that for a selected window size, the same BF is estimated using the SW approach (see BF for days 175, 176 and 177). However, BF varies temporally using the CW scheme (see BF for days 175, 176 and 177). The above table also indicates that cumulative rainfall depth of *in-situ* observations and corrected CMORPH remains the same (198.1 mm) for the SW approach.

### 4.3. Inverse Distance Weighted Interpolation

Studies by Dirks *et al.*, Hsieh *et al.*, Kong and Tong, Kurtzman *et al.*, Li *et al.*, and Wu *et al.* [39,40,41,42,43,44] showed that in general IDW produces equally good representation of spatially distributed rainfall as compared to methods such as Kriging or regression. Consequently, in this study the IDW interpolation is applied following Equations (5) and (6):

(5) $$R_{p}=\overset{N}{\underset{i=1}{\sum }}w_{i}R_{i}$$

(6) $$w_{i}=\frac{\frac{1}{d_{i}^{\propto }}}{\sum_{i=1}^{N}\frac{1}{d_{i}^{\propto }}}$$

where $R_{p}$ is the unknown rainfall depth (mm), $R_{i}$ is rainfall at known stations (mm), N is number of rainfall stations, $w_{i}$ is distance weight, $d_{i}$ is distance from the unknown location (*i.e.*, centre point of grid element) to a rainfall station and ∝ is distance power. In this study a power of 2 is applied following Goovaerts, Zhu and Jia, Lloyd, and Lin and Yu [45,46,47,48] to minimize rainfall interpolation errors at a daily time scale.

Pixel-based bias estimates for the selected bias removal scheme are spatially interpolated using IDW to yield a grid based BF’s distribution that covers the study area. The uncorrected CMORPH field is multiplied by the bias field to result in bias corrected CMORPH estimates. This method resembles the local-biased correction algorithm developed by Seo and Breidenbach [49].

### 4.4. Cross Validation

To assess results of IDW interpolation, cross validation described by Bivand *et al*. and Burrough and McDonnell [50,51] and recommended by WMO [52] is applied. This procedure involves withholding one gauge at a time and calculating the bias field without this particular gauge. This procedure is repeated systematically for all ten gauges with the validation dataset selected from the remaining data used in the earlier step. This method ensures that the gauge under consideration is selected once. The efficacy of the adopted technique is evaluated by whisker plots, in which the interpolated BF’s at the withheld gauge are compared against the corresponding *in-situ* BFs.

### 4.5. Overall Assessment of Bias Corrected CMORPH Estimate

The uncorrected CMORPH field is multiplied by the bias field to result in bias corrected CMORPH estimates. The overall performance is evaluated by Taylor’s diagram [53] that explains the statistical comparison between the time series of rain-gauge station (*i.e.*, bench bark), uncorrected CMORPH rainfall, bias corrected CMORPH and CMORPH version 1.0 (*i.e.*, test samples). It concludes how fair test samples relate to the bench mark. The performance of the proposed technique is assessed by statistical indicators such as RMSE, SD and r.

## 5. Results and Discussions

### 5.1. Sequential Window (SW)

**Figure 3:**
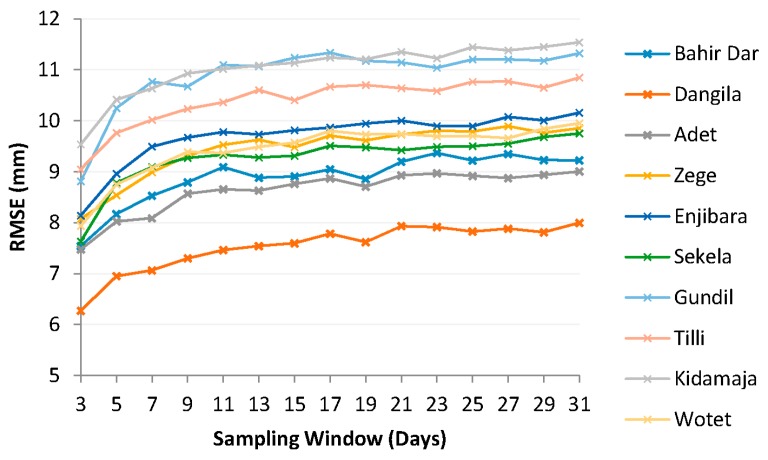
Shows result of sequential window analysis at different *in-situ* stations.

Figure 3 shows results for the SW approach. The performance of this approach is evaluated by RMSE between *in-situ* and satellite observations. Figure depicts RMSE of daily rainfall differences considering different window sizes. It shows that in general RMSE increases with increase in window size. RMSE increases considerably from a sample window of 3 days to a window of 7 days. For larger window sizes RMSE gradually increases with, generally smallest increases at very large (>15 days) windows sizes.

**Figure 4:**
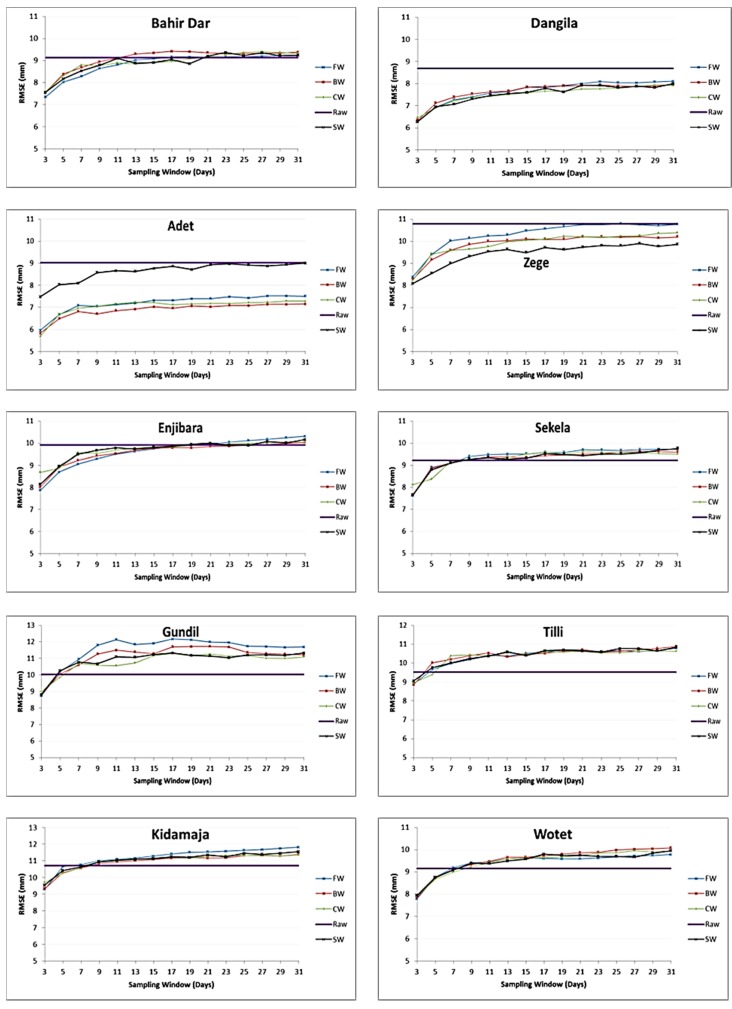
Results of FW, BW and CW schemes by the moving window approach.

This indicates that systematic errors in the SRE do not increase substantially for windows sizes larger than 7 days and thus effects of random errors only have little effect on the accumulated error. Few stations, e.g., Dangila and Bahir Dar, show that RMSE reduces for window size of 19 days. It is found that RMSE between uncorrected CMORPH estimates and *in-situ* observations may increase after application of sampling window. Figure 4 shows that at Gundil and Tilli stations, RMSE reduces till a sampling window size of 5 days. A similar trend is observed for the Sekela, Kidamaja and Wotet stations where RMSE increases after applying a 7 day sampling window. This may be due to over-adjustment of the uncorrected CMORPH estimates.

### 5.2. Moving Window (MW)

Figure 4 shows results for the MW approach. It indicates that for all three schemes (*i.e.*, BW, FW and CW), RMSE increases consistently with increased window sizes. Differences in RMSE for respective window sizes only are small and graphs do not show much deviation. In a few instances RMSE for a certain window size is larger than at increased window size. Examples are for CW scheme (for instance at 7 day window sizes at Bahir Dar and Enjibara stations respectively) and FW scheme (11 day window size at Gundil station). However, steadily increasing, smooth RMSE curves with comparatively lower values are observed for BW. Therefore, BW is selected and considered hereafter for further discussion and comparison with SW approach.

For the Dangila, Adet and Zege stations, a sampling window of any size produces better results than the uncorrected CMORPH data. For the Gundil and Tillli stations, it is found that a 5 day sampling window gives similar results as the uncorrected CMORPH data. It is also indicated that further progression in sampling window size would produce inferior results compared to use of raw CMORPH data. A similar trend is observed for 7 day sampling windows for three other stations (Sekela, Wotet, Kidamaja). It is found that an increase in window size will not always reduce RMSE. Therefore, an optimum window size of 7 days is selected from this analysis. This length is chosen for the following reasons:

(i) Habib *et al.* [37] also selected a 7 day sampling window to reduce the sampling variability of CMORPH data for the hydrological applications in the upper Gigel Abbay catchment, Ethiopia.
(ii) Analysis on window sizes of 3, 5, 7, …, 31 days shows that the RMSE values for daily rainfall differences levels off after 7 days (see Figure 4). Similar results were observed for the SW approach (see Figure 3).

### 5.3. Selection of Sampling Window Approach

**Figure 5:**
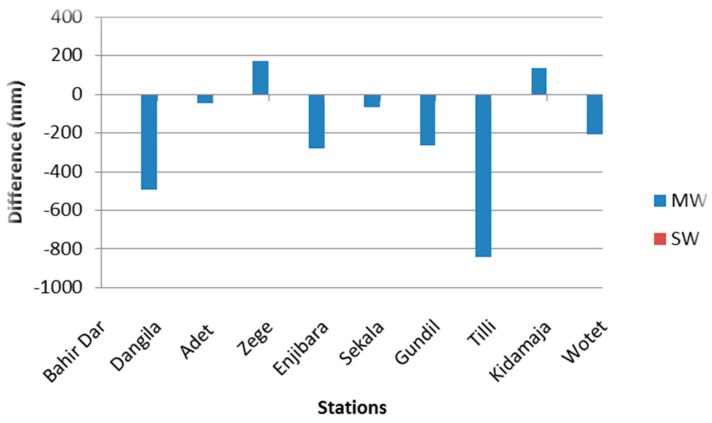
Bar plot showing accumulated difference between gauge observations and (i) 7 days MW approach (blue fill); (ii) 7 days SW approach.

We preferred SW over the MW approach as the RMSE stabilizes in SW analysis (see Figure 3). Also, in general, a lower RMSE is observed at all the stations (except Adet) by applying the SW approach. Figure 5 shows a bar chart with cumulative differences between gauge observations and corrected rainfall for 7 day MW and SW sampling windows. The analysis period is from 2003 to 2010.

It is found that cumulative rainfall depth of *in-situ* observations and corrected CMORPH remains the same for the SW approach. Therefore, a bar plot drawn for the SW approach does not show any difference in Figure 5. Large differences of cumulative rainfall depth were observed for the MW approach (such as 830 mm in the case of the Tilli gauging station). Therefore, we selected a 7 day SW approach for cross validation and correction of CMORPH satellite data.

### 5.4. Cross Validation

**Figure 6:**
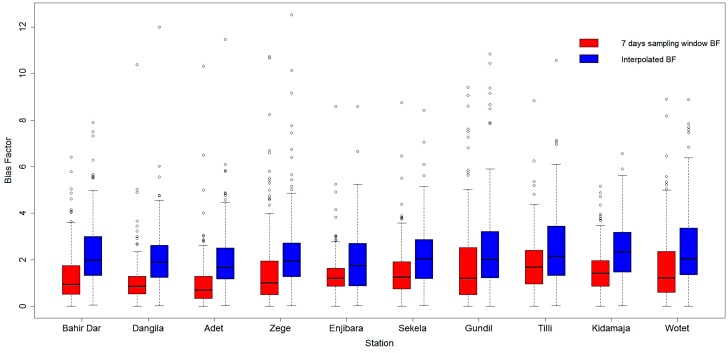
Whisker plot showing comparison of BF’s obtained from: (i) 7 day sampling window (red fill) and (ii) IDW interpolation (blue fill). The horizontal line inside the box depicts the median, the top and bottom boundary of the box depicts the upper and lower quartile (25% and 75%, respectively) while the whisker shows the maximum and minimum values excluding outliers. Outliers are shown by individual points (greater than 1.5 times the upper quartile). The *x*-axis denotes the rain gauge stations. The analysis period is from January 2003 to December 2010.

We tested the performance of the applied IDW scheme against 7 day sampling windows at each rain gauge station. Figure 6 shows the statistical summary of BFs at each withheld rain gauge station. It is found that for both the cases the lower whisker is at the same level (BF = 0), with no outliers found in the lower quartile. For the upper quartile, wider whiskers and high value of outliers is observed for the IDW method at all the stations (except Sekela, where the BF value of interpolated outliers is lower than the sampling window). The wide range of whiskers show the high variability of BFs for the IDW method.

It is also indicated that for each rain gauge station, the mean value for the IDW scheme is higher than with the sampling window method. For a 7 day sampling window, the minimum and maximum average value of BFs were found for the Adet (0.99) and Kidamja (1.85) stations, respectively, with an average BF value of 1.44 considering all the stations. In the case of the IDW method, the minimum and maximum average value of BFs were found for the Enjibara (1.88) and Gundil (2.49) stations, respectively, with an average BF value of 2.23 considering all the stations.

The average median value was found as 1.15 and 1.98 mm for the sampling window and IDW methods, respectively. For sampling windows, the minimum and maximum median value of BFs was found for the Bahir Dar (0.69 mm) and Dangila (1.68 mm) stations, respectively. The minimum and maximum mean values for the IDW method were found for the Adet (1.67 mm) and Kidamaja (2.34 mm) stations, respectively.

In general, the mean and median values of BFs for the interpolated scheme are slightly higher than with the sampling method. The wide range of BFs obtained from the interpolated scheme suggests that the temporal variability of BF is incorporated. It shows that the applied interpolation scheme is reliable and may be further used for correction of CMORPH data.

### 5.5. Evaluation of CMORPH Estimates

**Figure 7:**
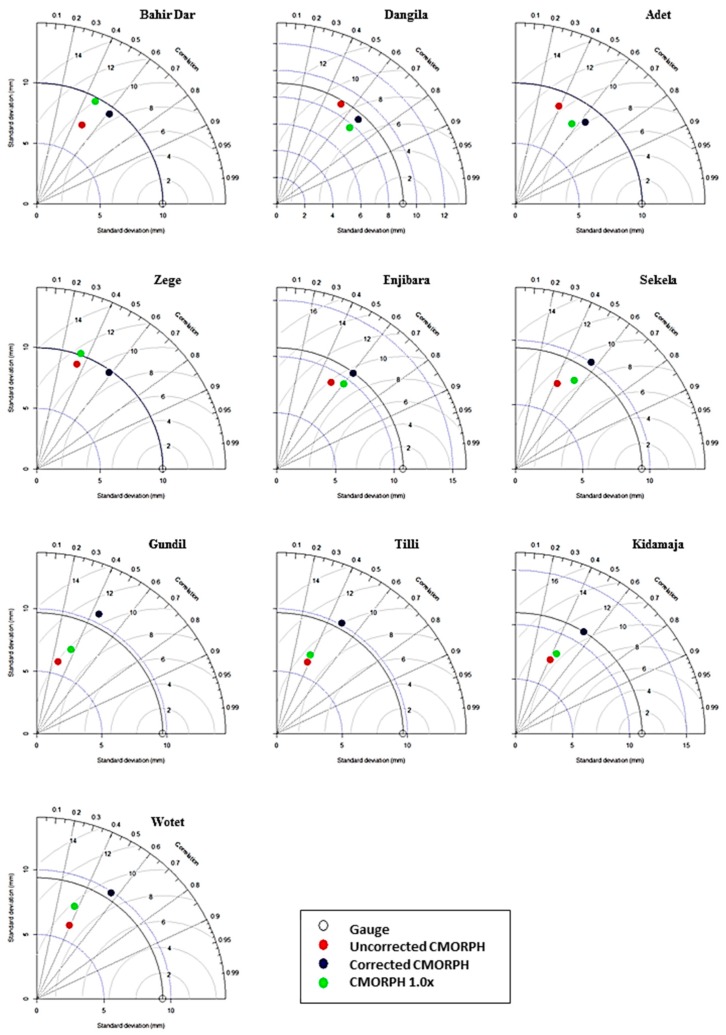
Taylors Diagram illustrating statistical comparison between uncorrected CMORPH, corrected CMORPH, CMORPH version 1.0 and the gauge rainfall (the bench mark). The azimuthal angle represents correlation coefficient; radial distance represents standard deviation (mm/day) of the rainfall time series and green contours represent RMSE (mm/day).

Findings on bias-corrected CMORPH estimates are illustrated in Taylor’s diagram [53], that depicts the statistical association between the test sample (uncorrected CMORPH, bias corrected CMORPH and CMORPH version 1.0) and the benchmark (gauge rainfall) [54]. Figure 7 sums up how well the test samples relate to the benchmark. The performance of bias-corrected CMOPRH estimate is evaluated in terms of RMSE, correlation and the standard deviation.

The relative attributes in terms of statistics for each test sample can be derived from Figure 7. The green isometric lines depict RMSE values between the sample and the benchmark patterns and are proportional to the point that shows the gauge data on the *x*-axis. The standard deviations of the test and benchmark patterns are represented by dotted and continuous black lines, respectively, and are proportional to the radial distance from the origin. The azimuthal angle represents the correlation coefficient value. Test sample patterns which agree well with the benchmark pattern lie close to the point marked on the *x*-axis. These patterns indicate a relatively high correlation and low RMSE [53].

The Taylor diagram is shown for all gauging stations. In general, better correlation is shown for biased-corrected CMORPH rainfall in comparison to uncorrected CMORPH and CMORPH 1.0 products. This is shown for all the gauging stations. Correlations vary from 0.25 to 0.52 (Gundil and Enjibara) and from 0.42 to 0.70 (Gundil and Dangila) for uncorrected and corrected CMORPH estimates, respectively. For CMORPH 1.0, the lowest (0.31) and highest (0.69) correlation is observed for the Zege and Bahir Dar stations, respectively. The largest increase in correlation (0.24) is observed for the Zege station where the correlation varied from 0.35 to 0.59 for the uncorrected and corrected CMORPH estimates. At all stations, RMSE between gauge observations and bias-corrected CMORPH estimates is reduced (except the Gundil and Tilli stations). The maximum change in RMSE is observed for the Adet station where it reduces from 10.5 mm to 8 mm for uncorrected and corrected CMORPH estimates, respectively. It is observed that our bias-corrected product gives lower RMSE than CMORPH 1.0 (see Taylor’s diagram for the Bahir Dar, Adet, Zege and Wotet stations). The SD of the uncorrected CMORPH estimate is found to be lower than the gauge SD at all the stations. For uncorrected CMORPH data, the lowest SD (6 mm) is observed for the Gundil station whereas the highest SD (8.9 mm) is observed for the Dangila station. It is clear from the figure that the SD of uncorrected CMORPH data improves after applying bias correction (see Figure 7: Sekela, Gundil, Tilli, Kidamaja and Wotet stations). In comparison with the gauge time series, SD of our bias corrected CMORPH is found better than CMORPH 1.0.

## 6. Summary and Conclusions

This study evaluated the effects of window sizes from 3, 5, 7, 9, …, 31 days on accumulated errors between time series of gauged daily rainfall estimates and daily CMORPH rainfall estimates. Moving window (MW) and sequential window (SW) approaches are tested for bias assessment and for calculating bias factors to correct the CMORPH estimates. The MW approach is tested for forward window (FW) backward window (BW) and central window (CW) to evaluate the effects of time integration on accumulated rainfall. RMSE is estimated to assess the error between the *in-situ* observations and CMORPH estimates. The objective was: (a) to evaluate bias between the *in-situ* (gauge) rainfall and satellite (CMORPH) data; (b) to identify the optimum window size for application of bias correction, and (c) to apply bias correction to CMPORPH data. Principle to the selection is that a long window size suppresses variability at smaller time scales such as ‘day by day’ variability whereas a short window size may result in low accumulation of rainfall depth thus, presumably, introducing very low (or very high) or even zero error in case of dry days. As such criteria for window selection are: (i) a window covering at least 3 days; (ii) a RMSE that does not increase pronouncedly with an increase of window size. For any analysis, rainfall detection by the satellite is assessed.

Bias in the CMORPH estimates is shown in Figure 4. The RMSE for uncorrected CMORPH data may be as high as 10.85 mm for the Kidamaja and Zege stations. It is found that in general RMSE increases with the increase in window size. The lowest value of RMSE is observed for a 3 day sampling window. It is also observed that increasing the window size will not always reduce RMSE. Steadily smooth curves that level off at a 7 day sampling window are observed for both the approaches. Therefore, this is selected as the optimum window length for correction of CMORPH data. It is shown that the BW scheme performs better than other schemes and that SW performs better than MW (Figure 5). Further, negligible differences between cumulative rainfall depths were observed for the SW approach. Therefore, SW of 7 days is selected for interpolation and cross validation. Figure 6 depicts that the mean and median values of BF’s for interpolated scheme are slightly higher than with the 7 day sampling method. The wide range of BFs obtained from the interpolated scheme suggests that any temporal variability of BF is incorporated. It suggests that the selected interpolation method may be applied for the correction of CMORPH data. The statistical relation between uncorrected CMORPH, corrected CMORPH, CMORPH 1.0 and gauge rainfall is shown in Taylor’s diagram (Figure 7). This figure shows that correlation is improved after applying the bias correction. The best improvement in correlation is observed for the Zege station. The maximum change in RMSE is observed for the Adet station where it is reduced from 10.5 mm to 8 mm for uncorrected and corrected CMORPH estimates, respectively. The high SD of the corrected CMORPH estimates shows that they capture the temporal variability present in the *in-situ* rainfall data.

The methodology is applied to a catchment where the density of rain gauge stations is sparse, so conclusions from this study may provide evidence for the utilization of CMORPH data for water resources assessment of the Lake Tan Basin. The outputs of this study will help hydrologists to understand the efficiency and application of CMORPH data in rainfall-runoff modeling.

## Figures and Tables

**Table 1:** BF calculation using MW and SW (FW, BW and CW schemes) approaches.

Day of Year 2003	Uncorrected CMORPH Rainfall (mm)	*In-Situ* Rainfall (mm)	3 Days Sequential Window Bias Factor	Corrected CMORPH Rainfall (mm)	3 Days Moving Window Bias Factor			Corrected CMORPH rainfall (mm)		
					FW	BW	CW	FW	BW	CW
172	5.3	32.0	2.75	14.59	2.75	8.41	3.93	14.59	44.55	20.80
173	8.7	6.7		23.95	2.00	3.93	2.75	17.38	34.15	23.95
174	0.6	1.5		1.65	5.27	2.75	2.00	3.16	1.65	1.20
175	8.7	27.8	3.28	28.49	3.28	2.00	5.27	28.49	17.38	45.84
176	6.5	54.0		21.29	2.86	5.27	3.28	18.60	34.25	21.29
177	14.8	16.5		48.47	1.42	3.28	2.86	20.96	48.47	42.36
178	22.9	56.0	1.33	30.53	1.33	2.86	1.42	30.53	65.54	32.43
179	13.5	0.0		18.00	0.41	1.42	1.33	5.58	19.12	18.00
180	8.3	3.6		11.07	1.30	1.33	0.41	10.80	11.07	3.43
Cumulative rainfall (mm)	89.3	198.1	-	198.1	-			150.1	276.2	209.3

## References

[1] Smith T.M., Arkin P.A., Bates J.J., Huffman G.J. (2006). Estimating bias of satellite-based precipitation estimates. J. Hydrometeorol..

[2] Tesfagiorgis K., Mahani S.E., Krakauer N.Y., Khanbilvardi R. (2011). Bias correction of satellite rainfall estimates using a radar-gauge product: A case study in Oklahoma (USA). Hydrol. Earth Syst. Sci..

[3] Habib E., ElSaadani M., Haile A.T. (2012). Climatology-focused evaluation of CMORPH and TMPA satellite rainfall products over the Nile Basin. J. Appl. Meteorol. Climatol..

[4] Vila D.A., de Goncalves L.G.G., Toll D.L., Rozante J.R. (2009). Statistical evaluation of combined daily gauge observations and rainfall satellite estimates over continental South America. J. Hydrometeorol..

[5] Ebert E.E., Janowiak J.E., Kidd C. (2007). Comparison of near-real-time precipitation estimates from satellite observations and numerical models. Bull. Am. Meteorol. Soc..

[6] Boushaki F.I., Hsu K.-L., Sorooshian S., Park G.-H., Mahani S., Shi W. (2009). Bias adjustment of satellite precipitation estimation using ground-based measurement: A case study evaluation over the Southwestern United States. J. Hydrometeorol..

[7] Rosenfeld D., Mintz Y. (1988). Evaporation of rain falling from convective clouds as derived from radar measurements. J. Appl. Meteorol..

[8] Joyce R.J., Xie P. (2011). Kalman filter-based CMORPH. J. Hydrometeorol..

[9] Xie P., Arkin P.A. (1995). An intercomparison of gauge observations and satellite estimates of monthly precipitation. J. Appl. Meteorol..

[10] Bitew M.M., Gebremichael M. (2011). Assessment of satellite rainfall products for streamflow simulation in medium watersheds of the Ethiopian highlands. Hydrol. Earth Syst. Sci..

[11] Bitew M.M., Gebremichael M., Ghebremichael L.T., Bayissa Y.A. (2011). Evaluation of high-resolution satellite rainfall products through streamflow simulation in a hydrological modeling of a small mountainous watershed in Ethiopia. J. Hydrometeorol..

[12] Haile A.T., Habib E., Rientjes T. (2013). Evaluation of the climate prediction center (CPC) morphing technique (CMORPH) rainfall product on hourly time scales over the source of the Blue Nile River. Hydrol. Process..

[13] Dinku T., Ceccato P., Grover-Kopec E., Lemma M., Connor S.J., Ropelewski R., Gutowski C.F. (2010). Validation of high-resolution satellite rainfall products over complex terrain in Africa. Int. J. Remote Sens..

[14] Hirpa F.A., Gebremichael M., Hopson T. (2010). Validation of high-resolution satellite rainfall products over complex terrain in Ethiopia. J. Appl. Meteorol. Climatol..

[15] Leander R., Buishand T. (2007). Resampling of regional climate model output for the simulation of extreme river flows. J. Hydrol..

[16] Shabalova M., van Deursen W., Buishand T. (2003). Assessing future discharge of the river Rhine using regional climate model integrations and a hydrological model. Clim. Res..

[17] Terink W., Hurkmans R.T.W.L., Torfs P.J.J.F., Uijlenhoet R. (2010). Evaluation of a bias correction method applied to downscaled precipitation and temperature reanalysis data for the Rhine basin. Hydrol. Earth Syst. Sci..

[18] Hay L., Clark M., Wilby R., Gutowski W., Leavesley G., Pan Z., Arritt R., Takle E. (2002). Use of regional climate model output for hydrologic simulations. J. Hydrometeorol..

[19] Rientjes T.H.M., Perera B.U.J., Haile A.T., Reggiani P., Muthuwatta L.P. (2011). Regionalisation for lake level simulation—The case of Lake Tana in the Upper Blue Nile, Ethiopia. Hydrol. Earth Syst. Sci..

[20] Haile A.T., Rientjes T., Gieske A., Gebremichael M. (2009). Rainfall variability over mountainous and adjacent lake areas: The case of Lake Tana Basin at the source of the Blue Nile River. J. Appl. Meteorol. Climatol..

[21] Rientjes T., Haile A.T., Fenta A.A. (2013). Diurnal rainfall variability over the Upper Blue Nile Basin: A remote sensing based approach. Int. J. Appl. Earth Obs. Geoinf..

[22] Haile A.T., Rientjes T.H.M., Habib E., Jetten V., Gebremichael M. (2011). Rain event properties at the source of the Blue Nile River. Hydrol. Earth Syst. Sci..

[23] Peel M.C., Finlayson B.L., McMahon T.A. (2007). Updated world map of the Köppen-Geiger climate classification. Hydrol. Earth Syst. Sci..

[24] Joyce R.J., Janowiak J.E., Arkin P.A., Xie P. (2004). CMORPH: A method that produces global precipitation estimates from passive microwave and infrared data at high spatial and temporal resolution. J. Hydrometeorol..

[25] Ferraro R.R. (1997). Special sensor microwave imager derived global rainfall estimates for climatological applications. J. Geophys. Res. Atmos..

[26] Ferraro R.R., Weng F., Grody N.C., Zhao L. (2000). Precipitation characteristics over land from the NOAA-15 AMSU sensor. Geophys. Res. Lett..

[27] Kummerow C., Hong Y., Olson W.S., Yang S., Adler R.F., McCollum J., Ferraro R., Petty G., Shin D.B., Wilheit T.T. (2001). The Evolution of the Goddard Profiling Algorithm (GPROF) for rainfall estimation from passive microwave sensors. J. Appl. Meteorol..

[28] Abdo K.S., Fiseha B.M., Rientjes T.H.M., Gieske A.S.M., Haile A.T. (2009). Assessment of climate change impacts on the hydrology of Gilgel Abay catchment in Lake Tana basin, Ethiopia. Hydrol. Process..

[29] Rientjes T.H.M., Haile A.T., Kebede E., Mannaerts C.M.M., Habib E., Steenhuis T.S. (2011). Changes in land cover, rainfall and stream flow in Upper Gilgel Abbay catchment, Blue Nile basin—Ethiopia. Hydrol. Earth Syst. Sci..

[30] Rientjes T.H.M., Muthuwatta L.P., Bos M.G., Booij M.J., Bhatti H.A. (2013). Multi-variable calibration of a semi-distributed hydrological model using streamflow data and satellite-based evapotranspiration. J. Hydrol..

[31] Wale A., Rientjes T.H.M., Gieske A.S.M., Getachew H.A. (2009). Ungauged catchment contributions to Lake Tana’s water balance. Hydrol. Process..

[32] Bastawesy M.E., Gabr S., Mohamed I. (2015). Assessment of hydrological changes in the Nile River due to the construction of Renaissance Dam in Ethiopia. Egypt. J. Rem. Sens. Space Sci..

[33] Enku E., Belachew M., Tilahun S., Steenhuis T. (2014). Biohydrology of low flows in the humid Ethiopian highlands: The Gilgel Abay catchment. Biologia.

[34] McCollum J.R., Krajewski W.F., Ferraro R.R., Ba M.B. (2002). Evaluation of biases of satellite rainfall estimation algorithms over the Continental United States. J. Appl. Meteorol..

[35] Xie P., Arkin P.A. (1996). Analyses of global monthly precipitation using gauge observations, satellite estimates, and numerical model predictions. J. Clim..

[36] Xie P., Arkin P.A. (1997). Global precipitation: A 17-year monthly analysis based on gauge observations, satellite estimates, and numerical model outputs. Bull. Am. Meteorol. Soc..

[37] Habib E., Haile A.T., Nazmus S., Zhang Y., Rientjes T. (2014). Effect of bias correction of satellite-rainfall estimates on runoff simulations at the source of the Upper Blue Nile. Remote Sens..

[38] Ines A.V.M., Hansen J.W. (2006). Bias correction of daily GCM rainfall for crop simulation studies. Agric. For. Meteorol..

[39] Dirks K.N., Hay J.E., Stow C.D., Harris D. (1998). High-resolution studies of rainfall on Norfolk Island: Part II: Interpolation of rainfall data. J. Hydrol..

[40] Hsieh H.H., Cheng S.J., Liou J.Y., Chou S.C., Siao B.R. (2006). Characterization of spatially distributed summer daily rainfall. J. Chin. Agric. Eng..

[41] Kong Y.F., Tong W.W. (2008). Spatial exploration and interpolation of the surface precipitation data. Geogr. Res..

[42] Kurtzman D., Navon S., Morin E. (2009). Improving interpolation of daily precipitation for hydrologic modeling: Spatial patterns of preferred interpolators. Hydrol. Process..

[43] Li B., Huang J.F., Jin Z.F., Liu Z.Y. (2010). Methods for calculation precipitation spatial distribution of Zhejiang Province based on GIS. J. Zhejiang Univ..

[44] Wu L., Wu X.J., Xiao C.C., Tian Y. (2010). On temporal and spatial error distribution of five precipitation interpolation models. Geogr. Geo-Inf. Sci..

[45] Goovaerts P. (2000). Geostatistical approaches for incorporating elevation into the spatial interpolation of rainfall. J. Hydrol..

[46] Zhu H.Y., Jia S.F. (2004). Uncertainty in the spatial interpolation of rainfall data. Prog. Geogr..

[47] Lloyd C.D. (2005). Assessing the effect of integrating elevation data into the estimation of monthly precipitation in Great Britain. J. Hydrol..

[48] Lin X.S., Yu Q. (2008). Study on the spatial interpolation of agroclimatic resources in Chongqing. J. Anhui Agric..

[49] Seo D.-J., Breidenbach J.P. (2002). Real-time correction of spatially nonuniform bias in radar rainfall data using rain gauge measurements. J. Hydrometeorol..

[50] Bivand R.S., Pebesma E.J., Gomez-Rubio V. (2008). Applied Spatial Data Analysis with R.

[51] Burrough P.A., McDonnell R.A., Lloyd C.D. (1998). Principles of Geographical Information Systems.

[52] Acharya N., Chattopadhyay S., Mohanty U.C., Dash S.K., Sahoo L.N. (2013). On the bias correction of general circulation model output for Indian summer monsoon. Meteorol. Appl..

[53] Taylor K.E. (2001). Summarizing multiple aspects of model performance in a single diagram. J. Geophys. Res. Atmos..

[54] Bhatti H.A., Rientjes T., Verhoef W., Yaseen M. (2013). Assessing temporal stability for coarse scale satellite moisture validation in the Maqu Area, Tibet. Sensors.

